# Advancements in Microfabricated Gas Sensors and Microanalytical Tools for the Sensitive and Selective Detection of Odors

**DOI:** 10.3390/s20195478

**Published:** 2020-09-24

**Authors:** Enric Perarnau Ollé, Josep Farré-Lladós, Jasmina Casals-Terré

**Affiliations:** 1 Department of Mechanical Engineering, Polytechnical University of Catalonia (UPC), MicroTech Lab, Colom street 11, 08222 Terrassa, Spain; josep.farre.llados@upc.edu (J.F.-L.); jasmina.casals@upc.edu (J.C.-T.); 2 SEAT S.A., R&D Department in Future Urban Mobility Concepts, A-2, Km 585, 08760 Martorell, Spain

**Keywords:** volatile organic compounds (VOCs), gas sensors, nanomaterials, microelectromechanical systems (MEMS), microfluidic devices, gas chromatography, lab-on-a-chip (LOC)

## Abstract

In recent years, advancements in micromachining techniques and nanomaterials have enabled the fabrication of highly sensitive devices for the detection of odorous species. Recent efforts done in the miniaturization of gas sensors have contributed to obtain increasingly compact and portable devices. Besides, the implementation of new nanomaterials in the active layer of these devices is helping to optimize their performance and increase their sensitivity close to humans’ olfactory system. Nonetheless, a common concern of general-purpose gas sensors is their lack of selectivity towards multiple analytes. In recent years, advancements in microfabrication techniques and microfluidics have contributed to create new microanalytical tools, which represent a very good alternative to conventional analytical devices and sensor-array systems for the selective detection of odors. Hence, this paper presents a general overview of the recent advancements in microfabricated gas sensors and microanalytical devices for the sensitive and selective detection of volatile organic compounds (VOCs). The working principle of these devices, design requirements, implementation techniques, and the key parameters to optimize their performance are evaluated in this paper. The authors of this work intend to show the potential of combining both solutions in the creation of highly compact, low-cost, and easy-to-deploy platforms for odor monitoring.

## 1. Introduction

In the last decades, monitoring of odors has been a relevant topic in applications such as air quality, environmental science, health care analysis, or forensic applications [1]. Moreover, humans’ olfaction has long played a significant role in industries such as wine-tasting, cuisine, perfumery, or product packaging [2]. In recent years, the unconscious perception of aromas has also shown to drive customers’ behavior and experience throughout many different applications [3]. Thus, the value of good smell has recently become a competitive factor for many industries to launch new products and services. In this context, new sensing devices and platforms that enable a fast, in-situ and real-time monitoring of odors are on the demand for current and future industrial applications [4]. Odorous species consist of volatile organic compounds (VOCs), which generally evaporate from solid or liquid sources at relatively low temperatures (i.e., ambient temperature). There exist hundreds of different VOCs that can originate unpleasant odors, and most of them can be detected by human’s olfactory system at concentrations that range from a few ppm (i.e., parts per million) to ppt (i.e., parts per trillion) trace levels [5]. Today, multiple gas sensors are commercially available for the monitoring of VOCs, and the selection of the most optimal device basically depends on each application [6]. Even though most of gas sensors today are still far to provide sensitivities closed to biological systems, recent studies show that the level of miniaturization of these devices can play a significant role to increase their sensitivity and overall performance [7,8]. In recent years, advancements in micromachining techniques have enabled the introduction of microelectric and mechanical systems (MEMS) for gas sensing applications as well [9,10]. Miniaturized gas sensors not only contribute to have more compact, portable, and low-cost devices but also enable the in-situ and real-time monitoring of compounds, which is a key advantage for odor monitoring applications [11]. Moreover, reported cases show that sensors incorporating micro- or nanomaterials in their structure (i.e., 0-D, 1-D, or 2-D composites) achieve significant improvements in their sensing performance as well. These structures own a high surface-to-volume ratio, which enable a better interaction with odorous species and target VOCs, in order to obtain higher sensitivities [12,13,14].

Nonetheless, a major concern of general-purpose gas sensors is their lack of selectivity towards odors and compounds of different nature [15]. Several strategies exist to improve the selectivity of single-based gas sensors, such as specially functionalized surfaces, doping of nanomaterials, temperature cycling, or the use of multicomposite materials [16]. However, these strategies tend to foster devices tailored to a very specific application, which compromise their modularity and flexibility of implementation. In order to tackle these problems, two well-known strategies exist to enhance the selectivity of gas sensors: (i) the use of cross-reactive sensor arrays with pattern recognition intelligence (e-noses) [17,18,19] or (ii) the use of chemical analytical devices, which force the separation of each individual compound in a mixture, employing long chromatographic columns or strong magnetic fields [20,21,22]. First of all, electronic noses have been on the spotlight of research for many years, due to their similarities to humans’ olfactory system and their good performance in the identification of complex odors and gas mixtures. Nonetheless, e-noses often present some limitations that hinder their wide-spread implementation, such as short lifetime, sensitivity to masking species, considerable dimensions, tedious operations, and high implementation costs, due to the nature and number of sensors employed by these systems, which can range from 2 to 40 units in some applications [23,24]. On the other hand, conventional analytical methods (i.e., gas chromatography/mass spectrometry) have been traditionally deployed in laboratory facilities, with bulky devices and trained professionals to run them. Thus, despite of the high selectivity offered by these systems, they are costly, lack portability, and provide poor flexibility. In this context, microanalytical tools represent a very good alternative to both, array-based systems and conventional analytical methods, for the correct discretization of multiple odors. These systems normally employ a single gas sensor for detection and have a very compact and portable size, which fosters the in-situ and real-time monitoring of VOCs. In the last decades, many efforts have been devoted to the miniaturization of conventional devices, such as gas-chromatography systems. Microgas chromatographs (µGC) try to incorporate all the key components employed in large-scale systems in a small and compact device, exploiting the use of new micromachining techniques. However, despite of the high performance showcased by these devices in some applications, they still present some concerns, such as complicated and tedious configurations, short lifetime, high-power consumptions, and high costs of implementation [25,26]. The price of current commercially available µGC devices range from 10 to 100 k€, which still limits their practical implementation in many applications [27]. For this reason, recent advancements in microfluidics have enabled to obtain new analytical tools for the discretization of individual VOCs in a mixture. These systems integrate a general-purpose gas sensor coupled with a functionalized microfluidic channel, in order to force the separation of analytes prior to detection [28,29,30]. Compared to other analytical tools, microfluidic-based devices avoid the use of long separation columns and complex electronics in their structure, offer a much simpler configuration, and can operate at room temperatures [31]. Hence, these new microanalytical devices have emerged as a very promising solution for the creation of practical, low-cost, and compact tools for odor discretization. This review intends to outline the potential of microfabricated gas sensors and new microanalytical tools in the creation of sensitive, selective, and easy-to-deploy platforms for the purpose of odor monitoring. In the first place, this work focuses on recent efforts done in the miniaturization of gas sensors for the detection of VOCs, as well as in the implementation of new nanomaterials to increase the sensitivity and overall performance of these devices.

The authors of this work intend to provide a general framework for researchers and nonexperts, with the principal families of gas sensors that exist today for the monitoring of VOCs. In addition, this work overviews the different types of nanomaterials that can be employed to detect odorous species, in terms of their properties, main characteristics, and implementation techniques. On the other hand, this review outlines recent advancements in microanalytical tools that can provide selectivity to stand-alone gas sensors towards VOCs of different nature. Special attention is paid to new microfluidic-based devices, as well as their synergies and differences with microgas chromatography systems, widely investigated in recent years. Since the segregation power of microfluidic devices is rooted on chromatographic columns, this work intends to identify the key components and parameters that determine the operating principle of both systems, as well as to discuss the optimum design requirements that enhance their selectivity.

## 2. Gas Sensors for VOCs Detection

**Figure 1:**
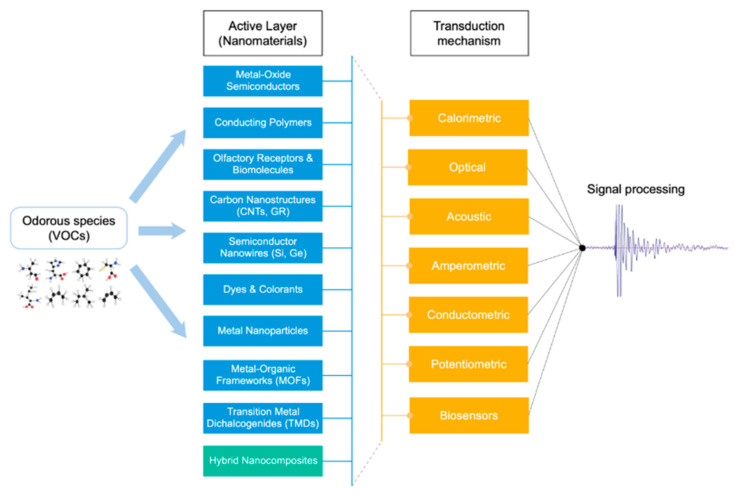
General framework with the different families of gas sensors that exist for the monitoring of VOCs. Gas sensors can be classified according to two basic principles: (i) the functional materials used to interact with the different compounds or (ii) the transduction mechanism employed for sensing.

Recent advancements in microfabrication techniques and nanomaterials have enabled to obtain increasingly sensitive and compact devices for the purpose of odor monitoring. This section intends to outline the different families of microfabricated gas sensors that exist for the sensitive detection of odorous species. It is generally accepted that VOCs are the main components in odors and aromas of different nature [32,33]. Thus, for the purpose of odor monitoring, there is the need of devices that can detect different VOCs at pretty low concentrations, ranging from a few ppm (i.e., parts per million) to ppt (i.e., parts per trillion) trace levels depending on the application. In general terms, gas sensors are devices that experience a change in one or several physical properties when they are exposed to vapor analytes [34]. They normally comprise a transducer and an active layer. The active layer converts a desired chemical interaction with VOCs into a change of its intrinsic properties (e.g., optical, acoustic, electrical, etc.), volume, or mass. The transducer is then responsible to trace these changes and convert them into a measurable electric signal, which relates to the analyte’s nature and concentration [34]. Hence, gas sensors can be grouped according to two basic principles of association: (i) the transducing mechanism being employed or (ii) the active layer used to interact with vapor analytes. Based on the transduction mechanism, gas sensors can fall into four general families: optical, electrochemical, gravimetric and thermal, or calorimetric devices. On the other hand, gas sensors can be classified based on the nature of the active layer they employ for sensing. Metal oxide semiconductors (MOS), polymers, carbon nanostructures, biomaterials, hybrid composites, and other nanomaterials are the six main categories of functional materials identified in the literature to interact with VOCs (see Figure 1).

### 2.1. Transduction Mechanisms

#### 2.1.1. Optical Devices

Optical gas sensors exploit a change in the optical properties of the sensing layer upon exposure to odorous species. Variations in light absorbance, fluorescence, polarization, color, wavelength, or reflectivity are generally recorded by a photodetector and converted into an electrical signal, which is proportional to the concentration and nature of analytes [35]. Optical devices that rely on reflectometric techniques have been widely reported for the detection of VOCs, such as Fabry–Perot or Mach–Zehnder interferometers and surface plasmon resonance (SPR) sensors [36,37]. These devices commonly make use of optical fibers to direct a light beam from a source to a detector, passing through a sensing membrane. Upon changes in the surrounding environment, this membrane undergoes a reversible change in its physical or chemical properties, which results in a modulation of the device reflectometric characteristics [38]. Regarding the performance of interferometric gas sensors, recent studies show that these devices can offer high sensitivities at a few ppm (e.g., 0–140 ppm [39]), limit of detection (LOD) in the ppb range (e.g., 140 ppb [40]), and response times of a few seconds. On the other hand, gas sensors that rely on fluorescence or colorimetric techniques have also been widely implemented for the detection of VOCs and odorous species [41,42,43]. These devices exploit the chemical interaction with target analytes to provide a color change of the sensing layer. They are normally constituted of a measurement chamber with a light source, an active sensing material, and a photodetector or camera to capture light modulation [43].

**Figure 2:**
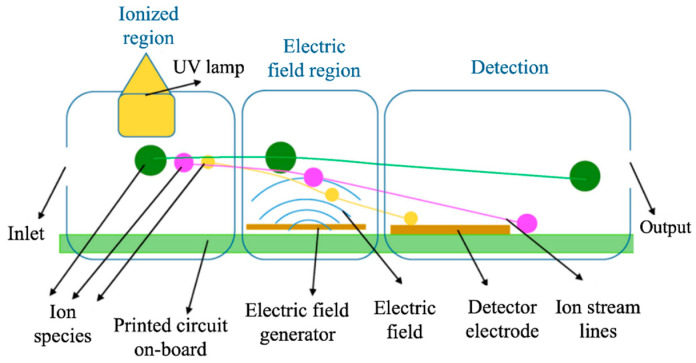
Schematic representation of different components in a PID optical gas sensor for the detection of different VOCs (color dots). Reprinted from ref [47]. Copyright 2018, Elsevier.

Colorimetric gas sensors can provide highly selective and discriminatory responses towards mixtures of various VOCs; hence, they are normally employed in e-noses for the detection of multiple odors and compounds [44]. Similar to interferometric devices, sensitivities of colorimetric gas sensors are generally at a few ppm and detection limits can be down to hundreds of ppb. However, they tend to offer longer response times (i.e., 2–12 min) [45]. Other common concerns of these devices are their poor sensitivity towards analytes with low reactivity and their lack of reversibility in some applications. There are other groups of optical devices that do not necessarily rely on the chemical interaction with odorous species. The performance of these devices can be rooted on two different working principles: the ionization of gas molecules or the absorbance of light [46]. Photoionization detectors (PID) belong to the first group of devices. They normally make use of an UV lamp to ionize all compounds in a gas mixture, which generate a signal proportional to the concentration of VOCs in a small measurement chamber (see Figure 2) [27]. Conventional PID normally offer fast response and recovery times, as well as high sensitivities towards small concentrations of VOCs (i.e., <50 ppm) [47]. In addition, recent efforts done in the miniaturization of these devices have contributed to their portability, low-cost implementation, sensitivities in the ppb range (e.g., 0–1 ppb), very low detection limits (e.g., 2–10 ppt), and response times in the order of milliseconds [48]. On the other hand, there are other sensors that base their functioning on the absorbance of polychromatic or infrared light, such as nondispersive infrared (NDIR) gas sensors [49,50]. When gases penetrate into the measurement chamber of these devices, they absorb light of a particular wavelength, which results in a unique spectra for each odor being analyzed [50]. This type of optical devices generally requires low energy to operate and is able to provide a certain degree of selectivity. However, conventional NDIR devices present some drawbacks such as bulky size (A~20–30 cm^2^), low sensitivities (i.e., 0–5000 ppm), high detection limits (i.e., LOD > 30 ppm), and high interferences with multiple species and compounds [46]. Nonetheless, recent studies show that a significant reduction in the detection limit of NDIR devices (i.e., LOD < 1 ppm) can be achieved by the use of optical fibers or interference correction factors [49]. In addition, photoacoustic devices (PAD) have emerged as a very promising alternative to improve the performance of NDIR and other optical devices [51]. Recent studies show a significant decrease in the LOD of PAD compared to traditional NDIR devices, which can be down to a few ppb (e.g., 10 ppb [51]). In PAD, VOCs are enclosed in a resonant acoustic chamber and sound waves are optically induced to each analyte based on the amount of light absorbed [52]. These devices normally employ a highly sensitive microphone to detect small acoustic signals or a piezoelectric element, which contributes to a reduction in the size of these devices, as well as their cost-effectiveness. In conclusion, optical gas sensors offer some attractive advantages for the monitoring of odors, such as high sensitivity, lower energy consumption, seamless connection to the communication network, and, in some applications, enhanced selectivity [53]. One of the main benefits of optical gas sensors is their high signal-to-noise ratio or, in other words, their immunity to environmental factors. For this reason, these devices are a good alternative for sensing in complicated environments, with the presence of flammable or explosive gases, very aggressive analytes, or strong electromagnetic fields [36]. Nonetheless, miniaturization of optical devices has been traditionally tedious and costly to achieve, due to the number of components needed in their operation. In this context, photo crystal (PC) optical sensors are raising a lot of interest in recent years, due to their small size (mm × mm), versality good performance towards the detection of VOCs [54,55]. PCs consist of a dielectric material with periodic micro- or nanopatterns in its structure that only allow specific wavelengths of light to propagate. Upon exposure to vapor compounds, these devices experiment a change in the refractive index or periodicity of the PC nanopatterns, which can be optically examined. In addition, recent advancements in nanomaterials and micromachining techniques have enabled to obtain one-dimensional PC structures (i.e., 1DPC), with acceptable sensitivities (i.e., LOD < 15 ppm), very fast response times (<2 s), and, in some applications, enhanced selectivity with a colorimetric-based response [56].

#### 2.1.2. Gravimetric Devices

**Figure 3:**
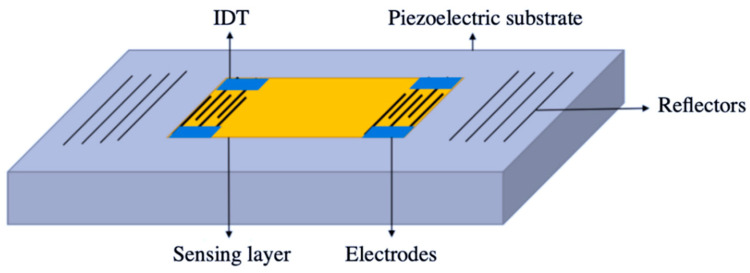
Schematic representation of a SAW gas sensor with two arrays or reflectors and a piezoelectric substrate. Reprinted from ref [7]. Copyright 2019, Sensors by MDPI.

Gravimetric or acoustic gas sensors can detect small mass-changes of the active layer when this is in contact with odorous species. These devices normally exploit the piezoelectric effect of crystals or microcantilevers, which resonate at specific frequency when they are subject to an acoustic wave [57]. Specific functional materials are normally coated on the surface of piezoelectric elements, in order to foster the absorption of VOCs, which then translates into a variation of the resonant frequency or amplitude of these elements [58]. Different groups of acoustic gas sensors are reported in the literature, based on the nature of the acoustic wave and vibration modes involved. Surface acoustic wave (SAW) sensors are one of the main groups of gravimetric devices based on piezoelectric crystals, which have been widely employed for the detection of odors [59,60]. These devices normally consist of two interdigitated transducer (IDT) responsible to generate and receive an acoustic wave that propagates on the surface of the piezoelectric crystal [61]. Figure 3 presents a schematic view of a typical SAW gas sensor with two transducers. Common implemented crystals in SAW sensors are lithium niobate (LiNbO_3_), gallium phosphate (GaPO_4_), and quartz [58]. One of the main advantages of SAW sensors is that generation, propagation, and detection of the acoustic wave are all confined in the crystal’s surface, which offers good opportunities for their miniaturization [62]. On the other hand, in bulk acoustic wave (BAW) the acoustic wave does not propagate on the surface, but through the interior of the piezoelectric crystal, which lowers the sensitivity of these devices compared to SAW sensors [57]. Quartz crystal microbalance (QCM) is one of the most reported BAW sensors for VOCs monitoring. In this case, a quartz crystal is sandwiched in between two electrodes, so that when an external electric field is applied, this generates a wave at the quartz’s resonant frequency [63]. Resonant frequencies of the crystal are inversely proportional to the its thickness; hence, thin crystal layers lead to higher frequencies and thus higher sensitivities of the sensor [64]. There exist other gravimetric devices that base their performance on the propagation of shared-horizontal acoustic waves through the piezoelectric crystal. This is the case of acoustic plate mode (APM), surface transverse wave (STW), and love wave (LW) sensors, which are generally implemented to detect VOCs in liquid solutions rather than gases [57]. Generally, SAW and BAW sensors have shown acceptable sensitivities, good response times, and suitability to be miniaturized in devices to just several micrometers [65]. Like many other gas sensors, the performance of gravimetric devices is strongly dependent on type of active layer employed. In [66], for instance, a SAW device with high sensitivities below 50 ppm and a LOD of 500 ppb is used for the detection of H_2_S based on a sol–gel CuO film, whereas in [67], a QCM sensor with a polymeric active layer is used to detect several VOCs, showing sensitivities below 500 ppm and a LOD of 5 ppm. However, acoustic gas sensors normally present elevated noise levels (~1–3 kHz), due to the high frequencies needed during operation, which can range from 200 [66] to 433 MHz [58] in some applications. Moreover, the performance of these devices gets compromised with the nature of the piezoelectric crystal and environmental factors (i.e., temperature and RH).

Another group of gravimetric devices widely employed for the monitoring of VOCs are the *flexural plate wave* (FPW) gas sensors. These devices make use of the so-called Lamb waves to cause a flexural deformation on the surface of a microcantilever or diaphragm, which is coated with a special sensing membrane [68]. Their working principal is pretty similar to the SAW sensors, i.e., IDTs are used to launch and receive an acoustic wave that propagates through a piezoelectric substrate. Thus, when vapor compounds are absorbed by the coating membrane, the device experiences a change in the oscillation amplitude or frequency, which is proportional to the analyte’s concentration and nature [57]. Nonetheless, FPW sensors incorporate an active cantilever whose thickness is much smaller than the acoustic wavelength, which causes the entire plate to oscillate with the propagation of the wave. As a result, FPW sensors are normally easier to miniaturize and can offer sensitivities one or two orders higher compared to SAW devices [69]. In addition, they can operate at lower frequencies (e.g., f~8 MHz) and still provide acceptable performances, which results in lower noise levels (i.e., <80 Hz) and less complicated electronics in their architecture [70]. The plate’s substrate is normally silicon functionalized with some piezoelectric material (e.g., zinc oxide (ZnO) or aluminum nitride (AlN)), so that an output AC electrical signal is obtained proportional to its vibration [71]. Recent advancements in micromachining techniques have shown outstanding results in the miniaturization of some acoustic devices that operate at ultrasonic frequencies, such as capacitive micromachined ultrasonic transducers (CMUTs) [72]. These devices consist of a flexible membrane coated with a specific sensing material and suspended over a static conductive membrane to create a small capacitor on top of an inert substrate. When the device is exposed to ultrasonic acoustic waves, the gap between membranes is modulated at the same frequency, which induces a constant change in the capacitance of the device [73]. The presence of vapor analytes results in a mass-change of the flexible membrane, which alters its modulation frequency, and therefore, the capacitance-change of the device over time. CMUTs have demonstrated promising features compared to other acoustic-based devices, such as (i) higher sensitivities (<ppb-level) and lower LOD (i.e., ppt range) [74]; (ii) small and compact design, with lengths of a few micrometer and widths in the nanometer scale, (iii) low operating frequencies (e.g., 4–14 MHz) [75,76], (iv) better signal-to-noise ratio (<10 Hz), or (v) low-costs of implementation [77,78].

#### 2.1.3. Electrochemical Devices

**Figure 4:**
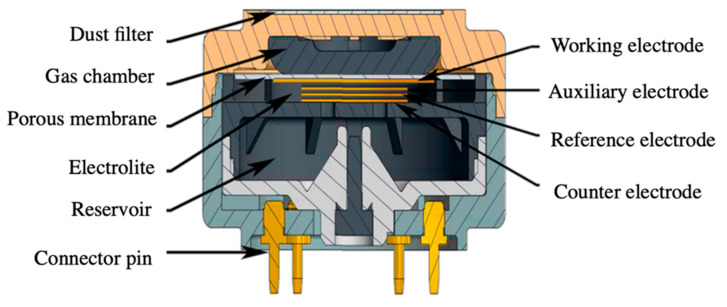
Schematic design of a standard amperometric gas sensor. Reprinted with permission from ref [84]. Copyright 2017, American Chemical Society (ACS).

Electrochemical gas sensors are maybe the most implemented devices used for the monitoring of odorous compounds [79,80]. Electrochemical sensors are able to detect small concentrations of VOCs, by assessing the electrical response of the device. According to the electrical signal being analyzed, electrochemical sensors can be divided in three main families: amperometric, potentiometric, and conductometric devices [81]. *Amperometric* gas sensors measure the current generated between a counter and working electrode in an electrochemical cell, which is proportional to the analyte’s nature and concentration [82]. The operating principle of these devices relies on a redox reaction at the surface of the working electrode, which results in a charge-transfer with the electrolyte in the cell [81]. The electrolytes are generally liquid solutions, in the form of mineral acids or organic solvents, although they can also be gel-like or gas depending on the application [83]. Amperometric devices normally count on three main parts: (i) a gas chamber, which incorporates one or several filters to control the inlet of gases; (ii) the electrochemical cell itself; and (iii) a reservoir for exhaust vapors or compounds during the electrochemical process (see Figure 4) [84]. Amperometric gas sensors offer some advantages compared to other devices, such as low power consumption and immunity to humidity changes. Moreover, they present acceptable sensitivity levels in the ppm range, long-term stability, and lifetime [84]. Besides, recent studies show that amperometric devices can exhibit a very fast response time (<5 s) under optimal conditions and active layer [85]. However, the selectivity of these devices is normally optimized to a reduced number of VOCs and their performance is highly sensitive to temperature changes [86].

In recent years, advancements in microfabrication techniques and the emergence of new electrolyte fluids have contributed to obtain low-cost and highly compact devices [87]. This is the case of new amperometric sensors incorporating room temperature ionic liquids (RTIL) as electrolytes, which have demonstrated unique electrochemical properties and very promising performances for the detection of VOCs, with sensitivities of a few ppm and LOD in the ppb range [88,89,90]. Potentiometric gas sensors are another group of electrochemical devices, which measure changes in the potential or electric field upon interaction with vapor gases. These devices have also been widely employed for odor monitoring purposes [91,92]. Potentiometric gas sensors can be deployed in a cell-based configuration similar to amperometric devices, using two or more electrodes in contact with an electrolyte. However, these devices do not require a current flow to operate and they normally employ solid-state electrolytes, such as yttria-stabilized zirconia (YSZ) [93]. Potentiometric gas sensors have shown good sensitivities upon different gases and hydrocarbons at sub-ppm levels [94]. Some studies also show that the combination of these devices in arrays can lead to even higher sensitives (i.e., 1–100 ppb) and response and recovery times of a few minutes [95]. Field effect transistors (FETs) are a well-known category of devices that fall into the category of potentiometric gas sensors. FETs are normally constituted of three metal contacts: source (S), gate (G), and drain (D), separated by an insulator, which normally acts as the active layer. Nonetheless, FETs sometimes can also use the S-D connection to place the active layer [96]. Catalytically active gate materials, such as platinum, palladium, or iridium can be used [94]; although liquid-ion gated FETs have also been widely investigated, especially in bioelectronic devices [97]. On the other hand, silicon-based substrates are commonly proposed for FET devices, due to its chemical inertness and resistance to high temperatures [94]. The working principle of these devices is pretty simple. When a threshold voltage is applied to G, an electric current is generated from S to D. Any gas reaction causing a change in the insulator or metal gate properties will result in a modulation of this current. Thus, FET responses are generally assessed by the change in gate’s potential needed to keep this current constant at a preselected target value. However, FET sensors require a strict control of the surrounding environment (i.e., temperature and humidity) and normally present high levels of noise and baseline drift [98]. Conventional FETs incorporate metal oxides in the active layer, which need high temperature to operate (e.g., 400–600 °C) and contribute to the power consumption of the device (~mW). Recent studies have proven the potential to use polymers or organic semiconductors (OFETs), which can operate at room temperature and show promising performances, with sensitivities down to a few ppm (<25 ppm), LOD in the ppb-level (>1 ppb), and very fast responses (~5 s) [99]. Conductometric or chemiresistive gas sensors are very likely the most implemented devices for the detection of VOCs, due to their simple design, easy-operation, low cost of fabrication, compact size, and facile miniaturization [81]. Conductometric devices measure the change in sensor’s conductivity or impedance upon exposure to vapor analytes [100]. These devices are commonly deployed using an active layer in between two or several metal interdigitated electrodes (IDEs), which are generally deposited on top of an insulating substrate, such as alumina, silicon, or quartz [101]. Some advantages of these sensors are their good sensitivity to a wide range of volatile compounds, as well as rapid response and recovery times at pretty low concentrations [102]. Conventional chemiresistors offer sensitivities at ppm-levels and response and recovery times that range from several seconds to a few minutes depending on the application [12]. However, recent advancements in micromachining techniques and nanomaterials have enabled to obtain devices with higher sensitivities and LOD at sub-ppm levels (e.g., 10 ppb) with just a few minutes of response (i.e., 2–3 min) [103]. Similar to FETs, chemiresistors have been traditionally deployed using metal oxides in the active layer, which normally require high temperatures to operate and contribute to the power consumption of the device (~mW) [104]. Recent advancements in micromachining techniques (e.g., screen or inkjet printing) have enabled to deploy new nanomaterials on top of chemiresistors (i.e., polymers, carbon structures, or hybrid composites), which operate at room temperature and can offer acceptable sensitivities (e.g., 1–100 ppm) and detection limits in the sub-ppm range (e.g., 800 ppb) [105]. Moreover, due to the simple and compact design of chemiresistors, these devices can be easily miniaturized and implemented onto flexible substrates, which shows great potential for their implementation in wearable applications [106,107]. On the other hand, similar to potentiometric gas sensors, main concerns of chemiresistors are their sensitivity to environmental factors (i.e., especially humidity), as well as their lack of selectivity, which might lead to the sensor’s baseline drift or ineffective performance in complex gas mixtures [108].

#### 2.1.4. Calorimetric Devices

**Figure 5:**
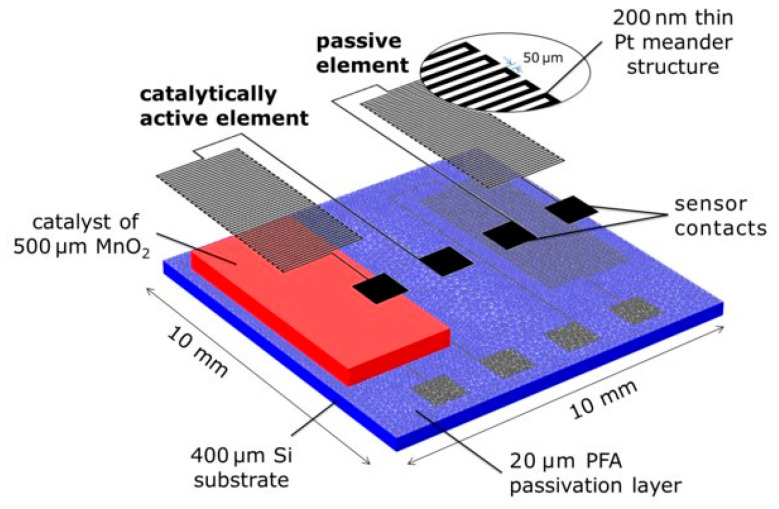
Schematic of a calorimetric H_2_O_2_ sensor. It consists of two Pt-meander structures: one passive and the other catalytically activated by a MnO_2_ layer. Reprinted with permission from ref [112]. Copyright 2017, Wiley-VCH GmbH.

Thermal or calorimetric gas sensors can also be employed for the monitoring of VOCs, although their application is normally limited to flammable or oxygen-containing species [109,110]. These sensors base their working principle on a catalytic exothermic reaction taking place at the surface of the sensor upon exposure to vapor analytes. Calorimetric devices normally employ two thermosensitive components, which convert enthalpy-changes at the surface of the sensor into an electric signal [111]. These components are generally deployed in the form of beads or using a metal-meander structure on top of a silicon-based substrate [112]. One of thermosensitive components is generally made active with a catalytic material coated on its surface, whereas the other remains inactive and it is set as reference. Noble metals (e.g., platinum (Pt) or palladium (Pd)) or metal oxide nanostructures (e.g., MnO_2_ or ZnO) have been widely reported as active catalytic materials in calorimetric gas sensors [113]. Figure 5 presents a novel microfabricated calorimetric gas sensor employing two Pt-based meanders, one acting as a passive element and the other catalytically activated by means of a MnO_2_ layer. Calorimetric sensors are generally used to detect explosion threshold limits of hydrocarbons and other VOCs in enclosed environments. Therefore, these devices are generally optimized to detect high concentrations of organic compounds (>1000 ppm) [114]. For this reason, calorimetric devices might not be suitable for the monitoring of odors, due to the high sensitivities normally required in this type of applications. Other common disadvantages of thermal gas sensors are short lifetime and high power-consumption rates of several Watts, since they normally operate at elevated temperatures of several hundreds of degrees Celsius [114]. Nonetheless, recent efforts in the miniaturization of these devices have contributed to obtain portable and small calorimetric sensors with enhanced performance [115]. The fabrication of microthermal sensors using MEMS technology has shown great potential and several advantages, such as very low power consumption (~mW), higher sensitivities, lower detection limits (e.g., 4–20 ppm) [116], and faster response times (e.g., t < 15 s) [114]. Nonetheless, the miniaturization of these devices can be tedious and costly to deploy, which is an important concern for their easy and practical implementation [117].

### 2.2. Functional Sensing Material

#### 2.2.1. Metal Oxide Semiconductors (MOS)

**Figure 6:**
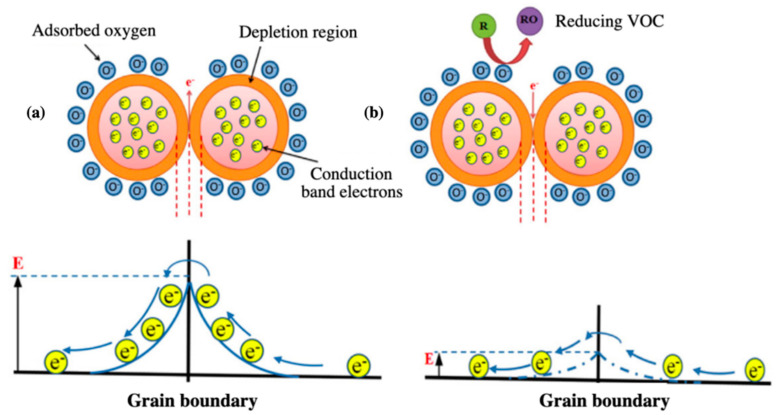
Model of the intergrain potential barrier of a n-type ZnO metal oxide semiconductor in (**a**) the absence of a target specie and (**b**) the presence of a reducing VOC (R). Reproduced and modified with permission from ref. [127]. Copyright 2015, Elsevier.

MOS are widely implemented as functional materials in chemiresistors [118,119] and potentiometric gas sensors [82,93]. The interaction between MOS and target analytes results in a redox reaction at the surface of the semiconductor, which translates into a change in its conductivity due to the formation or removal of oxygen molecules (i.e., O^2−^ and O^−^) [120]. Depending on the semiconductor employed, two main groups of MOS exist: n-type MOS (e.g., TiO_2_, ZnO, SnO_2_), which undergo an increase in conductivity when in contact with a reducing gas and a decrease in conductivity when in contact with an oxidizing specie, and p-type MOS (e.g., NiO, Mn_3_O_4_, and Cr_2_O_3_), which experience the opposite behavior [121]. Figure 6 represents the intergrain boundary behavior of a typical n-type MOS in the absence and presence of a reducing VOC. Compared to other sensing materials, MOS offers great stability, durability, and high sensitivity to small concentration of analytes (< ppm levels). In order to achieve greater sensitivities (< ppb levels), MOS are generally decorated with metal particles or other compounds, such as polymers, to conform hybrid composites [122]. Moreover, many reported cases state that the sensitivity and performance of MOS can be tuned by controlling several parameters, such as their composition, shape, morphology, doping levels, surface area, humidity, and operating temperature [123]. Among these parameters, MOS structures with large surface areas and small volumes have shown significant improvements in the detection of VOCs. Within this context, recent advancements in fabrication methods have enabled to deploy MOS nanocomposites and thin films of just a few nanometers thick (i.e., 1D and 2D structures), which have contributed to obtain increasingly sensitive, fast, and compact devices for odor monitoring [124]. Metal oxide nanostructures in the form of nanofibers, nanorods, or nanotubes are gaining a lot of attention in recent years, due to their unique properties and morphology. In [16], for instance, highly ordered and porous TiO_2_ nanotubes are fabricated for the detection of different VOCs. The inner diameters and lengths of the tubes were in the range of 110–150 nm and 2.5–2.7 μm, respectively. The nanotubes provide a higher surface area and a better interaction with analytes, which results in pretty high sensitivities (i.e., ~95% sensor response between 0 and 300 ppm). Another example are In_2_O_3_ nanobricks obtained in [125], showing lengths of 100–200 nm and width of 50–100 nm, which perform a high and uniform response between 100 and 500 ppb of NO_2_, at pretty fast response and recovery times (i.e., 114 and 49 s, respectively). Common methods for the synthesis of these materials are sol–gel or hydrothermal techniques. Besides, MOS nanostructures are normally deposited on top of rigid substrates using techniques such as spin coating, dip coating, drop-casting, screen printing, or electrochemical anodization [126]. On the other hand, some traditional concerns of MOS are the elevated temperatures needed during operation (150–600 °C), their cross-sensitivity towards organic and inorganic species, their difficult implementation onto flexible substrate, and the possible influence of some environmental factors, such as relative humidity (RH), in their sensing performance [123]. In addition, MOS-based sensors normally lack of selectivity towards multiple compounds. Traditional methods used to increase the selectivity of MOS mainly rely on the modification of its intrinsic properties, such as the utilization of special dopants or fillers, functionalized surfaces, or the use of temperature cycling [16]. However, recent studies show the potential of some MOS nanomaterials (e.g., TiO_2_ or In_2_O_3_), which provide enhanced selectivity towards target VOCs, while the intrinsic properties of the sensing layer remain untouched. In addition, most of these new nanocomposites have the ability to operate at low temperature, which reduces the power consumption of MOS-devices during operation, while still ensuring high levels of sensitivity and an overall good performance [125].

#### 2.2.2. Polymeric Materials

**Figure 7:**
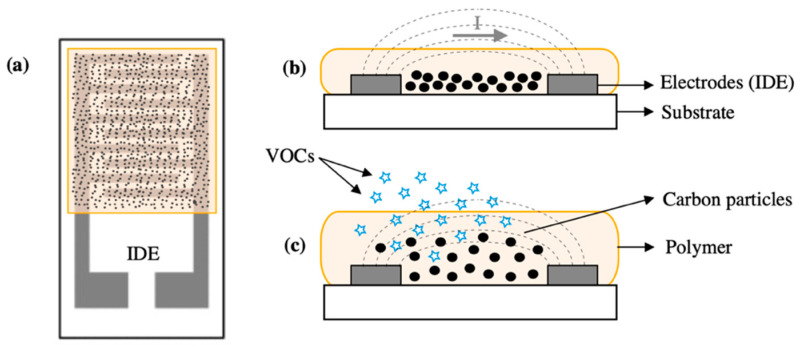
Working principle of an IDE-based gas sensor with a hybrid polymer composite: (**a**) top view of the gas sensor and the active layer; (**b**) representation of the polymer matrix with carbon fillers and its electrical characteristics prior to detection; and (**c**) polymer swell-effect due to the adsorption of VOCs, altering the distribution of fillers and overall impedance/conductivity of the composite [134].

Polymers are a group of functional materials that have attracted much interest for gas sensing applications, due to their inherent advantages such as low-cost implementation, good mechanical properties, easy synthetization, low energy consumption, miniaturization capabilities, and good response and recovery times. Polymers have been widely implemented in chemiresistors [128] and potentiometric or organic field effect transistors (OFETs) [96]. Intrinsic conducting polymers (CP) are normally chosen for the monitoring of VOCs. Similar to MOS, CP experience a change in their conductivity when they are exposed to vapor analytes. Even though MOS normally have higher sensitivities, CP present an attractive alternative to metal oxides due to their ability to operate at room temperature, which contributes to much lower power consumptions of the device [129]. CP are normally synthetized by chemical or electrochemical oxidation of their corresponding monomer and have conjugated π-electron systems in their structure, which make them conductive [130]. Some of the most typically CP implemented for gas sensing applications are: PANI, PEDOT, PPy, PTs, PA, and PPV. Common CP-based gas sensors have detection limits of about several ppm (i.e., <100 ppm) and response times in the order of minutes [129]. Better conductivity, controllable structure, and tunable properties of CP can be achieved by doping or functionalizing the organic structure, which is highly beneficial to obtain better performances [131]. Moreover, in recent years, 1D- or 2D-CP nanostructures have proven to increase the performance of this type of gas sensors. In [132], for instance, CP nanowires (<100 nm) are deployed as the active layer in a chemiresistive sensor array, using a cost-effective nanoscale soft lithography. The fabricated sensors show pretty high sensitivities upon exposure to different VOCs between 150 and 2000 ppm and LOD below 50 ppm. In addition, sensors exhibit short response (15–20 s) and recovery times (50–60 s), which is recorded to be 10 times faster than other CP gas sensors [133]. Insulating polymers (IP), such as PDMS, have also been employed in the literature for the detection of VOCs [134]. This structures are intrinsically nonconductive, but can be combined with CP or other fillers (e.g., carbon nanostructures, metal particles, metal oxides, etc.) to create hybrid composites with semiconducting properties [135]. Figure 7 schematically shows a hybrid polymer composite with carbon nanofillers and its behavior upon exposure to VOCs. Both, CP and IP hybrid composites have been widely used as sensing layers in multiple transduction devices, such as amperometric, chemiresistors, FETs, optical, or acoustic gas sensors [136,137,138]. Hybrid composites are generally preferred due to their better sensing performances compared to single polymeric layers, reaching detection limits in the ppb range (<1 ppm) and response times of just a few seconds (e.g., 2–3 s) [139]. In [140], for instance, the functionalization of a PANI-based gas sensor with graphene allows to decrease 10 times its detection limit (from 10 to 1 ppm). Finally, organic semiconductors (OSCs) are another group of polymers that have recently been employed in the form of nanocomposites for the effective detection of VOCs in both, OFET [141] and chemiresistor devices [142]. OSCs can be implemented as thin or ultrathin films, crystals, or nanofibers and have inherent semiconducting properties, which translate into certain conductivity levels upon the variation of some external factors (e.g., electric field, temperature, or photoexcitation). Following the same principle, when OSCs are in contact with target VOCs, they experience a variation in their semiconducting properties, which results in a measurable change of the electrical properties of the device (e.g., drain current in FETs) [143]. Poly(3-hexylthiophene) (P3HT) is one of the most investigated OSC polymers used for gas sensing applications, showing high sensitivities at a few ppm (<10 ppm) and ultrafast response and recovery times (i.e., 1–2 s) [142]. Besides, recent studies show other OSCs in the form of nanocomposites with even higher sensitivities, detection limits in the ppb range (e.g., 100 ppb), and also fast responses (e.g., 3–7 s) [144]. In conclusion, one of the main advantages of polymeric materials (i.e., CP, IP, and OSCs) is that they can be easily miniaturized into micro- or nanostructures, by employing new micromachining techniques, such as electrochemical deposition [139], drop casting, screen printing [145], soft lithography [132], micromolding [135], dip- or spin coating [96], which have enabled to deploy micro- and nanofilms onto target substrates. Owing to high surface-to-volume ratios, these micro- and nanopolymeric films offer a better interaction with target analytes, which contribute to high sensitivities and performances of gas sensing devices. In addition, one competitive advantage of polymers is that they can be easily deployed onto flexible substrates, which make them very suitable for wearable and flexible applications [145]. Nonetheless, pure polymeric materials present some disadvantages, such as poor stability, susceptibility to environmental factors, and shorter lifetime [128]. In addition, polymers generally get saturated upon exposure to high concentrations of analytes or multiple compounds [137]. For this reason, as it was mentioned before, the implementation of hybrid nanocomposites is widely recommended in the literatures to increase both, the properties and sensing performance of stand-alone polymers in the detection of different VOCs [146].

#### 2.2.3. Carbon Nanostructures

Recent advances in nanotechnology have enabled the introduction of zero-dimensional (0D), one-dimensional (1D), and two-dimensional (2D) carbon-based nanomaterials for the sensitive detection of multiple odorous species. Among them, carbon-nanotubes (CNTs) and graphene (GR) are probably the most implemented nanostructures used for the detection of VOCs. Carbon-based nanocomposites offer excellent characteristics for gas sensing applications, such as high thermal, mechanical, and electrical properties, good semiconducting behavior, and high surface-to-volume ratios, among other advantages [147,148]. For this reason, CNTs and GR have been widely employed in devices using different transduction mechanisms, such as optical [149], acoustic [150], conductimetric [151], or potentiometric [152] gas sensors. In addition, due to the biocompatibility of carbon nanomaterials, they have been widely employed as an immobilization layer in biosensors, between organic molecules and other functional materials, such as metal oxides or conducting polymers [153,154,155]. Both nanomaterials offer high sensitivities (ppb levels), low detection limits, good stability, and fast response times towards multiple analytes. Moreover, due to their intrinsic physical properties, CNTs and GR can be easily deployed onto both, rigid and flexible substrates [156]. Other advantages are their low cost of fabrication, excellent compatibility with other nanomaterials, as well as their ability to operate at room temperature [157]. Nonetheless, the implementation of pure carbon-nanomaterials may present some drawbacks, such as low affinity to some species, difficult manipulation, poor selectivity, long recovery times, and high sensitivity to fluctuations of humidity and other ambient conditions [147]. In order to improve the performance and properties of CNTs, their structure can be functionalized by means of chemical processes. Typical techniques found in the literature for the functionalization of carbon nanomaterials are hydroxylation or carboxylation of the carbon structure with selected acid solutions [158,159]. In addition, CNTs can also be functionalized with decorated particles or combined with other nanomaterials (e.g., polymeric films) to conform hybrid composites with enhanced characteristics [160,161,162]. One clear example of this can be found in [163], where the performances of pristine and functionalized CNTs are compared. This study concludes that functionalized CNTs provide 2–3 times higher sensitivities and a significant reduction in response and recovery times (i.e., ~12 and ~70 s, respectively). Regarding GR nanosheets, their structure can be chemically modified to obtain graphene oxide (GO) or reduced-graphene oxide (RGO), which provides ultrasensitive 2D or 3D composites with enhanced properties [164]. Recent studies show that functionalized GR nanostructures can achieve very low detection limits (~1 ppm to 6 ppb) and response and recovery times in the order of seconds (<100 s) [165]. The presence of oxygenated functional group on GO or rGO offer wide opportunities for their functionalization and make them highly hydrophilic, which explains why GO/rGO composites have been widely employed as active layers in humidity sensors [166]. However, the functional groups of these nanosheets facilitate the absorption of gas molecules into their structure, which enhances their sensitivity towards species, such as NH_3_, NO_2_, H_2_S, and multiple VOCs [167]. Hybridization of the GO/rGO structure with other nanoparticles or composites is also recommended in the literature to increase the performance of these materials, achieve better selectivity towards multiple species, or improve their mechanical properties [168]. Compared to CNTs, GR-nanosheets are generally produced more economically and present a better mechanical robustness, which enables their easy-transportation and implementation in complicated setups [166]. There are several techniques proposed in the literature for the synthesis of a single or multiple layer GR-nanosheets, such as micromechanical and chemical exfoliation, CVD, and other less explored methods, such as unzipping CNTs or the synthesis of graphene-like polyacyclic hydrocarbons. After the synthesis of GR, GO and rGO composites can be easily prepared by selected chemical processes, such as constant oxidation, the so-called Hummers method or electrochemical treatments [169]. On the other hand, CNTs can be fabricated and deposited on top of selected substrates making use of different methods. The growth of CNTs can be achieved by several techniques, such as arc-discharge, laser-ablation, or CVD [170]. After these processes, CNTs normally come with a number of impurities that need to be eliminated. Common purification activities rely on oxidation, the application of high temperature, or washing with acid solutions. CNTs can be then deployed onto target substrates using several techniques, such as drop-casting, electrophoresis, dip coating, or inkjet printing methods [105]. Despite the good properties of CNTs, they normally come highly entangled by strong van der Waals forces and tend to aggregate, which might compromise their sensing performance [171]. For this reason, a good dispersion solvent, such as isopropyl alcohol (IPA) or chloroform is generally used for the dispersion of CNTs prior to their implementation [172]. On the other hand, GR can be produced by chemical or micromechanical exfoliation of graphite, epitaxial growth, or CVD as well [155]. In addition, single or modified graphene are normally coated on top of target substrates by spin coating or dip coating techniques [152].

#### 2.2.4. Biological Composites

**Figure 8:**
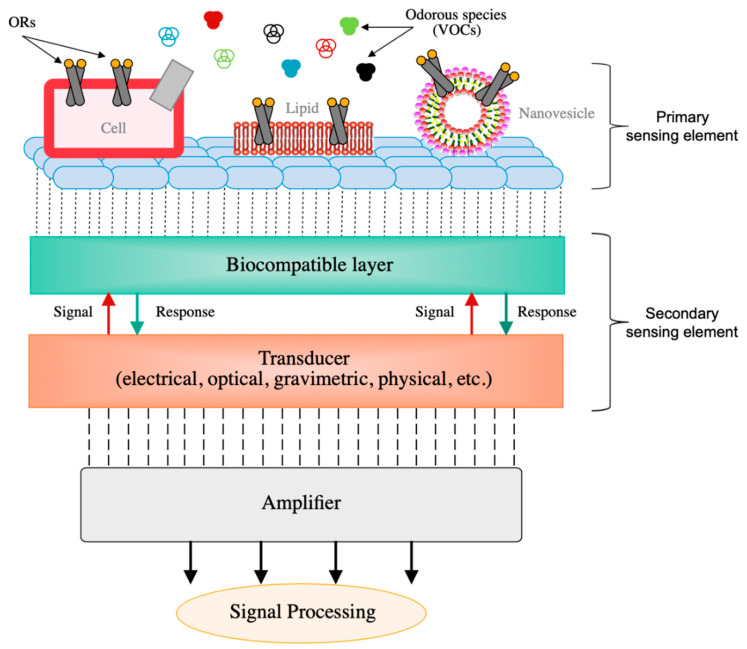
Schematic representation of a typical bioelectronic sensor device for the detection of odorous species (VOCs). These devices are generally constituted of a primary sensing element (i.e., biological element) and a secondary element used to capture and amplify the responses of bioreceptors [153]. Olfactory receptors (ORs) can be deployed onto these devices by means of cells or tissues, lipid layers, and nanovesicles [178].

Recent advancements in biotechnology and genetic engineering have enabled the implementation of olfactory receptors (ORs) and biomolecules as functional materials in biosensors, which try to mimic humans’ olfactory system in the detection of odorous species and VOCs. Olfactory receptors can be deployed in the form of cells or tissues, isolated OR-proteins, and nanovesicles [173]. Cells carrying multiple olfactory receptors are the most common configuration employed as a recognition element. Bacterial and yeast cells are some common examples of cells used [174,175]. Implementation of isolated OR-proteins or peptides instead of whole cells has been intensively researched due to their potential to scale down bioelectronic devices and achieve better sensitivity and selectivity rates [176]. An active field of research is on the application of odorant-binding proteins (OBP) directly from the sensory glands of insects and other vertebrates, which has shown greater performances compared to human-based ORs [177]. According to literature, there are three general methods for the immobilization of ORs on top of transducers, i.e., specific binding by antibodies or peptides, covalent binding through chemical reactions, and physical adsorption [173]. Nonetheless, the immobilization of isolated ORs onto a solid substrate is being a tedious and complicated task, and they normally constitute very unstable structures [178]. Recent studies show great potential to individual OR-proteins bonded using nanovesicles. Nanovesicles provide similar stability to cell-based configurations, but enhanced selectivity and sensitivity due to the incorporation of just selected olfactory receptors in their structure [179]. In biosensors, ORs and biomolecules normally act as primary recognition element. These elements are then immobilized on top of a biocompatible layer, which acts as a secondary sensing element to amplify small bioelectrical signals and achieve higher sensitivities [180]. In Figure 8, a schematic view of a typical bioelectronic device is presented, and the primary and secondary sensing elements can be distinguished. Biocompatible nanostructures such as polymers, MOS, or carbon nanomaterials are commonly used for this purpose [97,181,182]. In addition, cellulose nanofilms (1–100 nm) have recently emerged as a very promising group of nanomaterial to be used in biosensors as interfaces or substrates, due to their unique properties and high surface-to-volume ratios, which make them suitable for biomolecules immobilization and interaction [183]. For the binding of biomolecules on top of these elements, various processes are reported in the literature, which range from physical methods, such as physisorption, electropolymerization, or retention in sol–gel matrixes, to chemical methods, such as covalent cross-linking [153]. Finally, multiple transducing technologies have been employed in biosensors, which range from electrochemical devices to optical and acoustic gas sensors [184,185,186]. Some of the main advantages of biosensors are their high sensitivity, as well as high selectivity of biomaterials, based on the type of VOCs to be detected [153]. Common biosensors perform high sensitivities in a range of a few ppm (e.g., 5–80 ppm) and LOD in the sub-ppm range (<1 ppm) [177]. Recent studies also show that with the efficient immobilization of biomolecules and proper optimization of design parameters, biosensors can reach detection limits down to a few ppt (<10 ppt), which make them ideal for very sensitive applications such as breath analysis [180]. In addition, these devices show rapid response times and can be miniaturized at a relatively low cost. Nonetheless, biomaterials present some important drawbacks such as low stability, short lifetime, lack of reproducibility (in some applications), tedious fabrication processes, and difficult long-term maintenance [178]. In addition, one major concern of biomaterials is that they normally require a well-preserved and isolated environment to grow and be functional, which might compromise their application [178].

#### 2.2.5. Other Nanomaterials

Some other functional materials have been also used in gas sensing devices for the monitoring of VOCs. Dyes or colorants, for instance, have been widely employed in colorimetric and optical gas sensors. The presence of target analytes in the environment triggers the appearance or change in color of these elements. Typical chemoresponsive indicators are (i) pH indicators that respond to acidity/basicity of analytes, (ii) metal salts that respond to redox reactions, (iii) metal-ion containing dyes, (iv) solvatochromic dyes, and (v) nucleophilic indicators [123]. Main advantages of these elements are the in situ visual detection of target species as well as high selectivity towards specific analytes and compounds. In addition, they are normally very cheap and easy to deploy and offer flexibility for customization. However, one of the main disadvantages of dyes is that they normally offer a single-use application, contributing to a poor reproducibility [187]. Metal nanoparticles (MNP) have been widely implemented as monolayer in conductometric and other gas sensors due to valuable advantages, such as large surface-to-volume ratio, room temperature operation, sensitivities in the order of sub-ppb levels, low-voltage operation, fast response and recovery times, tolerance to humidity, and possibility to be deployed either on rigid or flexible substrates [188]. Some typical MNP implemented for gas sensing applications are Pt, Pd, Cu, Ni, Au, and Ag, which normally range from a few to hundred nanometers thick. One major advantage of MNP is that they normally present high selectivity towards specific gases or species. Pd, for instance, has the ability to change its physical, mechanical, or electrical properties upon exposure with H_2_ [189]. Other examples are the implementation of Ag for NH_3_ sensing [190], Au for alkanethiol sensing [191], Cu and Ni nanostructures for inulin sensing [192], or Pt or Pd for the detection of combustible gases. Thus, MNP are generally presented as good candidates to be employed in sensor array systems, for the sensitive detection of multiple VOCs in a mixture. Nonetheless, the implementation of pure metal nanoparticles as an active layer is still limited, mainly due to their elevated costs. For this reason, they are commonly employed as fillers in hybrid composites to increase the performance of other sensing materials, such as metal oxides, polymers, or carbon structures [193,194,195]. Pure semiconductors, such as silicon (Si) or germanium (Ge) nanowires, have been also used as sensing materials in FET and conductometric devices. Semiconductors are popular due to their compatibility with electronics and because doping or functionalization of their structure is a mature activity [196]. Moreover, they are very compatible with other nanomaterials, such as metal oxides or carbon nanostructures. Common techniques for the synthesis of Si nanowires include CVD, pulse laser deposition (PLD), thermal evaporation, and reactive ion etching, among others [123]. However, even though the sensing performance of Si nanowires has shown very promising results, they still present some challenges in the detection of nonpolar VOCs. Si composites, such as silica aerogels and films (SiO_2_) have also shown very good sensing capabilities. Nonetheless, silica is intrinsically nonconductive and presents weak mechanical properties, which hinder their implementation in conductometric devices for instance. For this reason, in some applications, SiO_2_ is filled with conducting polymers, carbon nanostructures, or MNP to overcome these challenges in the form of hybrid composites [197].

Metal–Organic Frameworks (MOFs) are another class of functional materials that have attracted much attention in recent years. MOFs can be defined as a porous crystalline structure constituted by the coordination of metal cations with organic ligands to form 1D, 2D, or 3D nanostructures [198]. They are a subclass of coordination polymers and offer some unique advantages, such as high porosity and surface area, high thermal stability, tunable adsorption affinities, and high compatibility with other gas sensing materials. MOFs have been used as an active layer in multiple applications, such as in optical (i.e., colorimetric, interferometers, or surface plasmon resonance devices) and acoustic gas sensors (e.g., SAW, QCM, or microcantilevers) [199]. Since most of MOFs are not electrical conductors, pure structures of these materials cannot be used directly in electrochemical devices. Nonetheless, recent efforts to combine MOFs with other conducting nanomaterials (i.e., carbon nanostructures or metal oxides) have boosted their implementation in chemiresistive devices, for instance [200]. Some studies show a considerable increase in the LOD (~100 times) and in response and recovery times (~2–3 min) of those sensors incorporating MOFs in the active layer, compared to their initial performances [98]. Transition metal dichalcogenides (TMDs) are another group of 2D sensing materials very attractive for the monitoring of VOCs. They are normally constituted of covalently bonded transition metal and dichalcogenide atoms arranged in the form of vertically stacked layers [201]. Typical TMDs, such as MoS_2_, WS_2_, ReS_2_, or MoSe_2_, offer large surface areas and unique electrical, chemical, and mechanical properties, which lead to high sensitivities (i.e., 1–1000 ppm), low detection limits (<10 ppb), high stability, and response and recovery times of a few seconds [165]. In addition, TMDs can operate at room temperatures and they are very suitable for the fabrication of flexible gas sensors [202]. Besides, they can provide certain selectivity towards target species, and their performance does not get compromised with high levels of relative humidity [165]. Common techniques for the synthesis of TMDs include mechanical exfoliation, electrochemical sonication, and CVD. Due to their unique semiconducting properties, TMDs are very suitable for conductometric or FET devices. Moreover, these nanomaterials also offer good optical properties, which make them attractive for optoelectronic applications as well. Nonetheless, TMDs normally provide long recovery times and they suffer from surface degradation, which might compromise their long-term stability [203]. For this reason, the structure of TMDs is sometimes tuned with special dopants, fillers, or nanomaterials (i.e., metal particle or metal oxides), which contribute to obtain tailored morphologies and achieve greater sensing performances.

In order to close this section, Table 1 intends to showcase the main differences between conventional gas sensors and newly developed microfabricated devices for the detection of VOCs. The objective of this table is to highlight the improvement in the performance and operation of these devices, by means of some representative examples found in the literature.

## 3. Microanalytical Tools for VOCs Discrimination

Chemical analytical methods have been widely employed in large-scale facilities for the discrimination of multiple VOCs in complex odors and gas mixtures. These strategies rely on the different structure and chemical composition of compounds, with the objective to force their individual separation and achieve their qualitative and quantitative recognition [208]. Common laboratory techniques used for this purpose are: gas chromatography (GC), mass spectrometry (MS), infrared spectroscopy (IR), or ion-mobility spectroscopy (IM). Among all these methods, GC and coupled systems (e.g., GC-MS) are probably the most implemented large-scale techniques in analytical chemistry for the discrimination of VOCs [209,210,211]. The segregation power of conventional GC-systems is determined by the interaction of VOCs between a mobile and a stationary phase. The mobile phase is generally injected in the form of a carrier gas (e.g., H, He, or N_2_), which is responsible to carry target analytes through a capillary column until they reach a final detector [212]. The capillary column is then coated with a stationary phase, strategically selected to foster the physical and chemical interaction with vapor compounds and force their separation [213]. Thus, the working principle of GC-systems rely on the different “retention times” that analytes spend inside the separation column, which depend on factors such as the nature of VOCs and the stationary phase or the operating temperature [214]. Even though conventional GC systems are highly precise and selective towards hundreds of different VOCs, they need to operate in big laboratory facilities, and normally require of sampling processes to collect, store, and transport gas samples directly from the source [215,216]. In addition, preconcentration activities are generally required to ensure the good performance and selectivity of these systems [217]. Apart from their lack of portability and bulky size, other disadvantages of conventional analytical systems are the high temperatures needed during operation, as well as their long operating times, which can be up to several hours [218]. In this context, many efforts have been devoted in the last decades to the miniaturization of conventional analytical devices. A wide range of portable and microgas analyzers are commercially available today for the selective detection of VOCs, such as the measurement device X-PID Series 9000/9500 from bentekk [219] or the 490 Micro-GC from Agilent [220]. Despite the portability and high selectivity provided by these devices, they are still quite difficult and expensive to deploy, which limits their applicability. Other commercial devices, such as FROG-5000 from Defiant Technologies are easier to handle and deploy, but they still come with high costs of implementation [221]. However, recent advancements in micromachining techniques and microfluidics have contributed to obtain increasingly compact, and miniaturized analytical tools, which foster the in-situ and selective monitoring of VOCs in a much cost-effective manner [222]. Moreover, these devices offer faster response and operating times, enhance the analysis of smaller volumes, and eliminate the risk of contamination, degradation, or loss of samples being analyzed.

### 3.1. Microgas Chromatographs (µGC)

Among all these new systems, microgas chromatographs (µGC) have been widely investigated in recent years, for the on-site and real-time discretization of VOCs [223,224,225]. Recent advancements in microelectromechanical systems (MEMS) have enabled to incorporate all relevant components of conventional GC-systems in a compact and portable device [225]. Thus, µGC normally include microfabricated components for injection (µ-injectors), separation (µ-columns), and detection (µ-detectors) activities. First of all, µ-injectors allow the introduction of small concentrations of analytes into the system with a selected carrier gas. They are normally constituted of a set of microchannels and one or several microvalves, which are activated based on different operation principles (e.g., magnetic, pneumatic, passive, or electromechanic) [223]. Similar to conventional methods, µGC normally employ microfabricated preconcentrators prior to injection, in order to purify gas samples, reduce detection limits (< ppb), and achieve better performances. One common concern of preconcentrators is that they generally require high temperatures to operate, which might compromise the correct operation of other µ-components in the system and contribute to higher power consumptions [226]. In addition, one of the most critical components in µGC is the microfabricated column. Similar to conventional devices, µ-columns are strategically coated with a stationary phase to force the separation of vapor analytes [227]. Nonetheless, compared to conventional capillary columns, µ-columns are several magnitudes shorter, noncylindrical, and normally microfabricated on top of planar substrates, using a chip-based configuration [228]. The separation performance of µ-columns will depend on the optimization of several factors, such as: (i) channel cross-section (e.g., rectangular, square, trapezoidal, or semicircular), (ii) channel design (e.g., circular or square spiral, serpentine, zigzag, radiator, or wavy), (iii) column typology (e.g., open, semipacked, or monolithic columns), (iv) substrate material, (v) stationary phase, (vi) operating temperatures, (vii) flow rate, and (viii) carrier gas [229]. Metal, glass polymers, and silicon-based materials are the most common substrates used in µ-columns, due to their good physical, thermal, and chemical properties [230]. Moreover, polymeric stationary phases, such as polydimethylsiloxane (PDMS) and its derivative, are generally preferred due to their good handling, chemical-inertness, and high porosity, which contribute to high separation performances, especially with nonpolar analytes [231]. Some of the most implemented techniques used for the coating of stationary phases onto µ-columns are static and dynamic coating, vapor deposition methods, electrodeposition, or packing [232]. Regarding column typology, semipacked columns have gained a lot of interest in recent years, due to their higher performances compared to common open channels [233]. Semipacked columns incorporate an array of micropillars embedded into the channel, which increases the contact surface between analytes and the stationary phase and contributes to higher separation efficiencies [234]. Another important feature of µ-columns is that they need relatively high temperatures to operate. However, compared to conventional GC columns, they normally require lower temperatures (i.e., <100 °C), which result in lower power consumptions of these elements. In addition, temperature programming strategies are actively proposed in the literature to foster the energy-efficient operation of µ-columns and ensure their compatibility towards all kinds of analytes [228].

**Figure 9:**
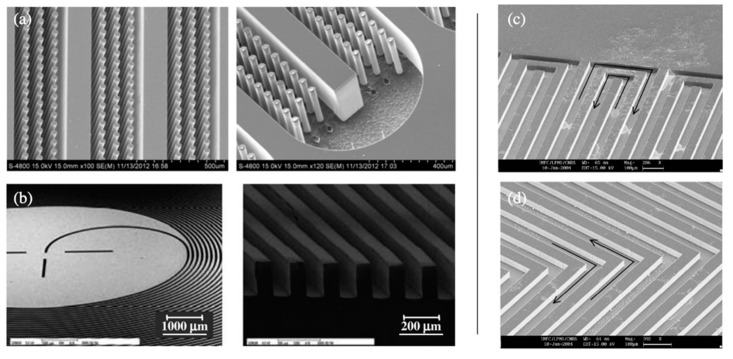
SEM images of different microchromatographic columns layouts on a chip-based configuration: (**a**) semipacked column with embedded microposts, (**b**) circular spiral, (**c**) square spiral, and (**d**) radiator. Reprinted with permission from ref. [234] (**a**), ref. [230] (**b**), and ref. [244] (**c**,**d**). Copyright 2013 Elsevier (part a), Copyright 2009 IEEJ (part b) and Copyright 2006 Elsevier (part c, d).

As it was stated before, µ-columns can adopt a large variety of channel cross-sections and designs. Some studies claim that serpentine channels have a greater performance than circular or spiral designs, for instance [235]. However, there is still not a clear consensus among researchers regarding the optimum channel layout for µ-columns. Figure 9 presents some of the most typical layouts of µ-columns investigated in the literature. Nonetheless, several studies show that the separation efficiency of µ-columns has a direct correlation with the geometrical properties of the channel, such as length and the so-called aspect ratio (depth vs. width) [236]. Generally speaking, columns incorporating long channels with high aspect ratios have proven better separation efficiencies. First of all, increasing channel depth contributes to greater volumes of the µ-column, which result in a higher sample capacity (i.e., maximum concentration of analytes that can be injected, without overloading the system) and flow-rates [237]. Second, reducing channel width fosters a better interaction between analytes and the stationary phase, which also contributes to their better segregation [238]. In addition, narrower channels enable to fabricate µ-columns with closer plates, which results in more compact devices or longer µ-columns within the same confined space. On the other hand, long channels allow higher flow-rates and force analytes to interact longer with the stationary phase, which normally leads to higher resolutions as well [236]. Nonetheless, it can be easily seen that the optimization of one-dimensional factor cannot be achieved without compromising the others [239]. Thus, the optimum length, width, and height are generally a trade-off between achieving high efficiencies and reaching acceptable response and recovery times. In recent years, comprehensive two-dimensional microgas chromatography (2D-µGC) has been actively proposed in the literature to improve the separation capacity and performance of standard µGC devices [240]. Moreover, 2D-µGC is a new microanalytical technique that couples a first-dimension column (D^1^) to a relatively short second-dimension column (D^2^), whose retention properties help to increase the number of compounds separated at a given analysis [241]. A micropneumatic or -thermal modulator (µTM) is normally employed at the interface between both columns, in order to trap analytes as they elute from the D^1^ and reintroduce them into the D^2^, by rapid heating and as a series of narrow pulses [242]. Recent studies show that higher performance can be achieved by using a two-stage µTM, where analytes are trapped and released in a two-stage process by applying low and high temperatures, respectively. This alternating heating–cooling process helps to avoid samples lost and incomplete trapping during thermal transitions in single-stage modulators [241]. Nonetheless, one common concern in µTMs is that they generally require high power intensities to operate. In order to tackle this problem and achieve greater performances, some studies suggest to employ a multichannel architecture with several D^2^ columns in parallel [243].

**Figure 10:**
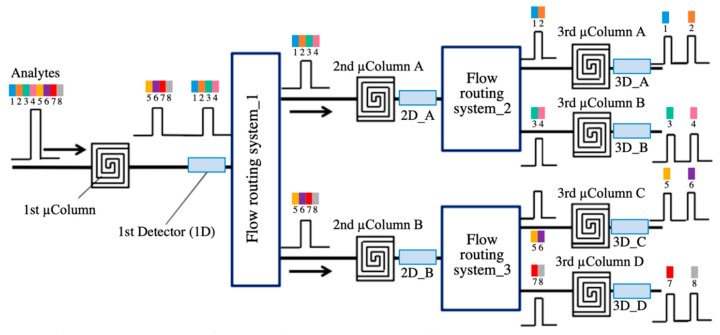
Schematic representation of an automated 3D-µGC system. It consists of a 1 × 2 × 4 channel adaptive configuration with three different levels of separation. The initial vapor mixture consists of eight different VOCs. After each separation column, there is a nondestructive detector connected to a computer-controller flow routing system that directs each vapor peak to the next column [246].

These systems incorporate a first detector at the end of the D^1^ column and a fully automated routing system, which directs the flow to one of the D^2^ columns based on predefined control algorithms. Thus, when an entire elution peak (i.e., analyte) passes through the first detector, this is sent to one of the D^2^ columns for further separation and final detection [245]. All D^2^ columns are independent from each other and normally present different properties (e.g., length, stationary-phase, operating temperature, etc.), which offers flexibility and optimal gas analysis according to the nature of each analyte. In addition, multichannel systems allow to reduce the length of D^2^ columns and separation times significantly. Moreover, they offer high scalability and simplified data analysis and avoid the use of high-power µTM for the injection of analytes into the system [246]. However, multiple dimensional µGC systems present complex and tedious configurations and time-consuming operations, which might compromise their easiness of use in many applications. Figure 10 shows the schematic representation of a multichannel µGC-system employing three dimensions for separation. Even though µGC-systems have proven performances close to conventional analytical methods, their practical implementation in real-world applications is still very limited. A major challenge of these systems is the interfacing of all µ-components in a single miniaturized and compact device [247]. According to literature, µGC can be deployed using both: (i) a hybrid configuration, where all µ-components are fabricated separately and manually assembled, or (ii) an integrated chip. Hybrid configurations are normally time consuming, expensive to deploy, and lead to prone errors and degradation of the whole system over time [248]. In addition, hybrid systems generally have large dimensions (i.e., dozens of cm^2^), which can compromise their easy handling and implementation. Even larger dimensions are encountered in systems employing multiple channels and separation µ-columns (e.g., 2D-µGC) [249]. Part of these problems can be solved if all µ-components are fabricated and integrated in a single microchip. However, a common problem in this type of configuration is the thermal crosstalk between components, which can compromise their operation and lifespan [250]. Microchip configurations are normally employed in commercial devices, while hybrid setups are mostly found for the purpose of research and investigation. Other common disadvantages of µGC-systems are the need of complex electronics for the seamless operation of all µ-components, as well as their complicated designs, which normally results in costly and tedious manufacturing processes. In addition, some of the µ-components used in µGC-systems compromise their miniaturization (e.g., carrier-gas tank), while others contribute to the high-power consumption of these systems (e.g., µ-columns, preconcentrators, or µTMs) [251].

### 3.2. Microfluidic-Based Devices

**Figure 11:**
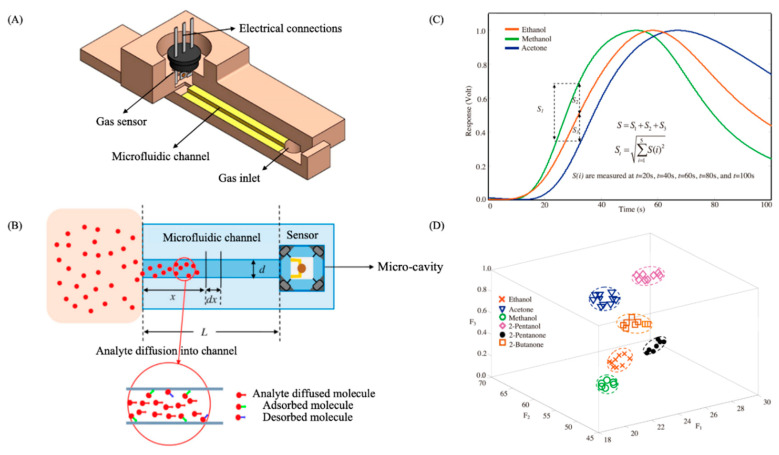
(**A**) Schematic diagram of the 3D-printed microfluidic device with a square-based cross-section. (**B**) Schematic representation of the diffusion and physisorption effect of gas molecules along the microfluidic channel (red dots). (**C**) Normalized transient response of the microfluidic-based device towards three different VOCs (ethanol, methanol, and acetone). The output signals are shifted onwards due to the selectivity provided by the microfluidic channel. (**D**) 3D feature space representation of six different analytes at eight different concentration levels (from 250 to 4000 ppm). Reprinted and modified with permission from ref. [254]. Copyright 2016, Elsevier.

In recent years, microfluidic-based devices have been introduced as a very promising alternative to µGC, for the selective identification of VOCs in binary, triple, or even more complicated gas mixtures [252,253,254]. These devices provide selectivity to a general-purpose gas sensor, by fostering the natural diffusion of analytes through a specially coated microfluidic channel (see Figure 11A) [254]. The working principle of these devices is rooted on chromatographic columns, employed in macro- and microanalytical tools. Nonetheless, microfluidic channels normally have lengths several magnitudes shorter (<10 cm), do not require a carrier gas tank, and can operate at room temperature [255]. Molecular diffusion and surface physisorption of gas molecules are two physical properties with considerable span among species. Microfluidic devices exploit the variation of these two parameters to control the transient flow of vapor analytes along the channel (see Figure 11B) [256]. Thus, the performance of microfluidic-based devices is dependent on the diffusivity and physical adsorption/desorption of gas molecules from and to the channel walls. The temporal variation of analytes concentration along the channel at isothermal and isobar conditions can be determined by this expression [257,258,259]:

(1) $$\frac{\partial C\left(x,t\right)}{\partial t}=D\cdot \frac{\partial^{2}C\left(x,t\right)}{\partial x^{2}}-\frac{\partial C_{S}\left(x,t\right)}{\partial t}$$

where *D* is the diffusion coefficient of each analyte in the mixture and $C_{S}\left(x,t\right)$ is the concentration of gas molecules physiosorbed into the walls of the channel. The previous equation is valid from channel depths or diameters ranging between 1 mm and 1 µm. For larger channel dimensions, diffusion of gas molecules along the channel is the most dominant parameter in the previous equation, so the term relative to physisorption vanishes to conform the free molecular diffusion equation [258]. By decreasing the channel depth, a larger number of gas molecules interact with the surfaces of the channel and the physisorption term gets proportionally more relevant. Nonetheless, the previous expression is unable to describe the diffusion processes in ultrathin channels (*d* < 1 µm), where other physical mechanisms should be taken into account [259]. According to literature, if a microfluidic channel with circular cross-section is considered, the concentration loss $C_{S}\left(x,t\right)$ due to the physisorption effect can be represented by the following expression [257]:

(2) $$C_{S}\left(x,t\right)=\frac{4C_{a}}{d}\cdot \frac{bC\left(x,t\right)}{1+bC\left(x,t\right)}\;$$

where $C_{a}$ is the number of the surface adsorption sites available per unit volume of the channel, d is the effective channel depth, and b is generally defined as the physisorption constant, which is directly related to the nature of analytes. Combining the physisorption expression to the diffusion equation initially stated, the so-called diffusion-physisorption equation can be formulated, which gives the change in analytes concentration over time and along the microfluidic channel [255,257]:

(3) $$\left(1+\frac{4C_{a}}{d}\cdot \frac{b}{{\left(1+bC\left(x,t\right)\right)}^{2}}\right)\frac{\partial C\left(x,t\right)}{\partial t}=D\cdot \frac{\partial^{2}C\left(x,t\right)}{\partial x^{2}}$$

In many experimental studies, both the concentration of analytes and b values are established to be less than the unit. Thus, the numerical value for bC(x,t) is normally much smaller than 1. Therefore, at low analyte concentrations the “diffusion-physisorption equation” can take the following approximate form:

(4) $$\left(1+\frac{4\alpha }{d}\right)\frac{\partial C\left(x,t\right)}{\partial t}\approx D\cdot \frac{\partial^{2}C\left(x,t\right)}{\partial x^{2}}\;$$

where α is defined as the adsorption coefficient ($\alpha =bC_{a}$), which is directly related to the physical interaction between gas molecules and the channel walls. From the previous Equation (4), it can be easily deducted that the physisorption effect of gas molecules increases, either by reducing the channel’s depth or raising the adsorption coefficient α [252]. For this reason, the selectivity and performance of microfluidic devices relies on the optimization of several factors, such as (i) channel’s geometry, (ii) coating of the channel walls, and (iii) environmental conditions (i.e., temperature and relative humidity) [254]. First of all, microfluidic devices normally incorporate straight channels of a few centimeters length, with either circular or square cross-sections [252,255]. Similar to chromatographic columns, design and geometrical properties of the channel are strategically selected to achieve the best selectivity, while ensuring acceptable response and recovery times [260]. Thus, length, width, and depth of the microfluidic channel play a significant role in the performance of these devices. However, in this case, the longer is generally not the better. Due to the small free diffusivities of vapor analytes inside the channel, long geometries would lead to long operating times. Hence, the performance of microfluidic devices is strongly dependent on the physisorption of gas molecules to/from the channel walls [254]. For this reason, high surface-to-volume (S/V) channels are recommended in microfluidic devices, which can be easily achieved by increasing channel’s width and reducing its depth or diameter [252]. On the other hand, nature, properties, and characteristics of the channel coating material also play a key role in the segregation power of microfluidic devices [253,261,262]. In general terms, those materials that foster a better physical adsorption between analytes and the channel walls have a positive effect on its performance. For this reason, chemically inert and highly porous materials, such as thin polymeric films, are commonly proposed for the coating of microfluidic channels [261]. Poly(3,4-ethylene-dioxythiophene):poly(styrene-sulfonate) (PEDOT:PSS) or Parylene C are some examples of polymers coated onto the inner surfaces of the channel. They can be deployed using well-known techniques, such as sputtering, chemical vapor deposition (CVD), or spin coating [262]. Nonetheless, other reported studies suggest different coating materials, such as metal oxides or single metal layers like gold (Au), due to its low reactivity values [252]. In addition, a common practice found in the literature is the use of coatings with multiple layers, in order to foster a better adhesion into the channel walls and increase their stability and overall performance [262]. In recent years, some studies have also demonstrated a correlation between the polarity of gas molecules and the channel coating, and the performance of microfluidic devices [262]. Generally speaking, if polarities are similar, there is a better interaction between analytes and the channel walls, which results in larger retention times and higher efficiencies [263].

**Figure 12:**
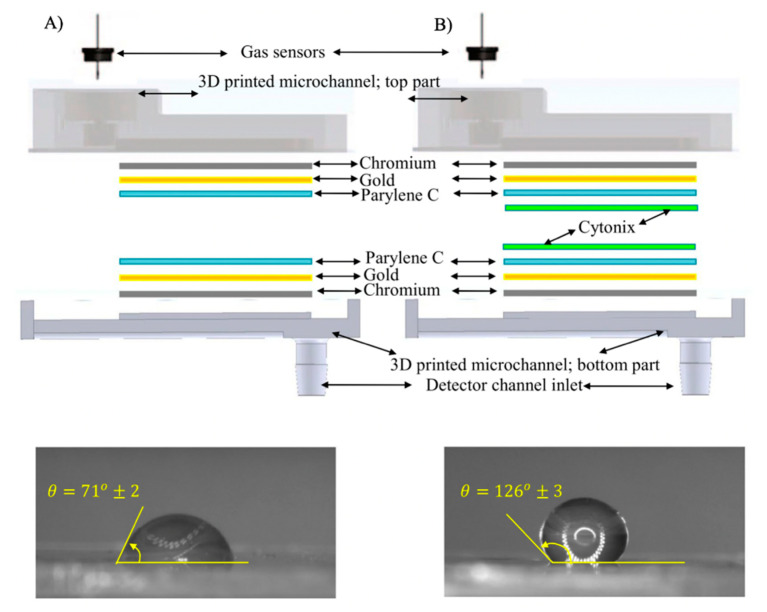
Multilayer coating of a microfluidic channel: (**A**) three-layer coating with chromium, gold, and Parylene C and (**B**) four-layer coating adding Cytonix, a highly hydrophobic material that enhances interaction with nonpolar analytes. Reprinted from ref. [262]. Copyright 2019, Springer Nature.

This effect is especially notorious with nonpolar analytes, while in the case of polar species, the coating polarity has lesser impact. This can be attributed to the higher diffusivities of polar gases compared to nonpolar ones, which results in shorter times of polar molecules to interact with channel inner surfaces. Thus, when it comes to the selection of the best coating material, nonpolar or highly hydrophobic coatings are generally preferred [264]. Figure 12 shows the changes in the polarity of the channel walls after employing a highly hydrophobic coating material. Besides, recent studies have proven that, in addition to the coating polarity, the roughness of the channel walls has also a direct effect on the performance and selectivity of microfluidic devices [253]. Several methods have been proposed in the literature to change the roughness of the channel, such as adding nanostructures or imprinted nanoparticles in its surface or making use of special mechanical engineering processes [265]. Finally, humidity and temperature fluctuations also have a negative impact on the performance of microfluidic-based devices. Particularly, changes in humidity can seriously compromise the selectivity provided by these devices. Some recent studies have shown that microfluidic systems fail to differentiate between several species or even between several concentrations of the same analyte, with slight fluctuations in relative humidity (~5%). In order to minimize this effect, reported cases suggest to incorporate a humidity control system, in order to remove its influence in the response of microfluidic devices [266]. In order to analyze the segregation power of microfluidic-based devices, the transient response of the sensor is normally assessed over multiple analytes. It has been demonstrated that changes in the analyte’s concentration alter only the amplitude of the sensor’s response, while different analytes also contribute to a small shift (i.e., onward or backward) of the response signal, due to the different interaction of each analyte with the microfluidic channel [252]. A common practice found in the literature is the normalization of sensor’s transient response between 0 and 1, in order to minimize fluctuations in signal’s amplitude, due to different analyte concentrations or sensor drift, and focus only on the selectivity provided by the microfluidic channel (see Figure 11C) [256]. Regarding data analytics, two or three features are generally extracted from the response pattern to represent analytes in a 3D or 2D feature space, which is used for comparison (see Figure 11D). The times in which the normalized response reaches 0.05 (tr) and 0.95 (tm) of its maximum value, together with the normalized response level at *t* = 120 s (Rf) are the three common features extracted from the sensor’s response [254]. Each VOC being analyzed defines a unique position in the feature space, which is shared by species or molecules of the same nature in the form of clusters. Similar to e-noses, microfluidic devices are normally trained, so that unknown species can be related to a certain group of analytes of known position in the feature space [256]. Besides, it has been proven that variations in analytes’ concentration has low effect in the conformation of these clusters; hence, different concentrations could hardly cause the misclassification of compounds [260]. However, fluctuations in environmental factors (e.g., relative humidity) have shown to compromise the representation of each analyte in the feature space significantly [266]. After obtaining raw data from the different measurements, several data-processing techniques are proposed in the literature for the purpose of odor identification [267]. These techniques normally rely on artificial intelligence tools, such as principal component analysis (PCA), independent component analysis (ICA), discriminant factorial analysis (DFA), cluster analysis (CA), partial least-squares analysis (PLS), k-nearest neighbor (KNN), or artificial neural networks (ANN), which foster the automatic identification of unknown species to one specific odor or group of chemicals previously trained [268].

## 4. Conclusions

In conclusion, this work has successfully showcased the potential of microfabricated gas sensors and new microanalytical devices, in the creation of sensitive and selective tools for odor monitoring. These tools represent a promising alternative to conventional analytical devices as well as array-based systems (e-noses) and open up a full window of opportunity for the practical and cost-effective monitoring of odors in multiple applications. In the first place, this review has presented the principal groups of microfabricated gas sensors that exist for the sensitive detection of VOCs. Based on their transduction mechanism, gas sensors can fall into four big families: optical, gravimetric, electrochemical, and calorimetric gas sensors. The principal advantages and drawbacks of each transducer have been reviewed in this work. Besides, the working principal and different typologies of these devices have been identified. In conclusion, advancements in micromachining techniques can contribute to obtain increasingly compact, light, flexible, and portable transducers for the monitoring of VOCs, which are key for the widespread implementation of gas sensors in odor-sensing applications. Second, this work has highlighted the different groups of nanomaterials that can be employed to interact with VOCs. These can fall into six main categories: metal-oxide semiconductors (MOS), polymers, carbon nanostructures, biocomposites, hybrid structures, and other nanomaterials. Advancements in micromachining techniques have enabled to come up with 0-D, 1-D, or 2-D structures, which can provide high levels of sensitivity. Owing to the high surface-to-volume ratios, nanomaterials provide a better interaction with target analytes, which results in a greater overall performance and optimum operation. In the past, these materials could not reach the performance of other high-power composites, such as conventional MOS. However, with the conformation of new micro- and nanostructures, MOS and other functional materials (i.e., polymers or carbon nanocomposites) are able to reach high sensitivities (i.e., < ppb levels), while still ensuring a low-cost operation. In addition, hybrid composites, combining two or more functional materials in their structure, have enabled to increase the sensitivity, stability, and overall performance of single nanocomposites in the detection of VOCs. Finally, bio-materials also showcase great potential in the sensitive detection of VOCs and odorous species. Despite the good performance and high sensitivity of bioelectronic devices, they require complex fabrication processes and need of very specific conditions to operate, which still hinder their scalability and easy implementation.

On the other hand, this work has reviewed recent efforts done in the conformation of microanalytical tools for the selective detection of VOCs. These tools could represent a good alternative to both, conventional analytical methods and electronic noses for the purpose of odor discrimination. In the area of microanalytical tools, microgas chromatographs (µGC) have been widely investigated in the last decades, due to their good selectivity provision and small and portable size. µGC force the diffusion of gas molecules along µ-columns, which are strategically coated and designed to foster their segregation. In order to optimize the separation efficiency of µ-columns, long channels with high aspect ratios (depth vs. width) are generally recommended. In general terms, those columns that foster a higher sample capacity and promote a better interaction between analytes and the stationary phase show greater performances. In addition, multiple-dimensions µGC systems, with two or more separation columns in parallel, have demonstrated to improve the selectivity and efficiency of single µGC significantly. Nonetheless, µGC systems need high temperatures to operate and require a carrier gas tank and complex electronics to control all the µ-fabricated elements in their structure (e.g., injectors, valves, preconcentrators, etc.). All these factors not only compromise the miniaturization and lifetime of these systems but also contribute to tedious and time-consuming configurations, difficult operations, and high-power consumptions.

For this reason, microfluidic-based devices have recently emerged as a very promising alternative to those systems, for the fast, versatile, and cost-effective discrimination of multiple VOCs in a mixture. Even though microfluidic devices are still far to provide the segregation of other analytical tool, these devices have recently proven good selectivity in samples with more than eight different analytes. Table 2 shows some of the main differences between microfluidic devices and other microanalytical tools widely investigated for selectivity provision. Microfluidic-based devices count on an optimized microfluidic channel, which is attached to a general-purpose gas sensor for detection purposes. Compared to µGC and other analytical methods, these devices can operate at room temperature without employing a carrier gas, which results in a more compact and portable design, low-cost fabrication, and simple and easy implementation. The segregation power of these devices relies on the free-diffusion of gas molecules along the channel, which tends to be rather small. Hence, in microfluidic channels, the physisorption of gas molecules with the channel walls is normally more relevant than diffusion to foster their good separation. For this reason, microfluidic channels are generally of a few centimeters’ length, straight, and designed to achieve high surface-to-volume ratios (width vs. depth). Moreover, recent studies show that the nature and properties of channel’s material coating has an important effect on the performance of these devices, especially with nonpolar analytes. Hence, the optimization of channels’ geometry, coating material, as well as a good control of environmental factors (i.e., temperature and relative humidity) are extremely important for the separation efficiency and performance of microfluidic-based devices. Finally, even though microfluidic devices are still far to reach the market and need of advanced technical development, the combination of these systems with new microfabricated gas sensors showcases great potential for the practical and low-cost monitoring of odors in future industry applications.

## Figures and Tables

**Table 1:** Characteristics and operating principles of common sensors for volatile organic compounds (VOCs) detection. Microfabricated devices are compared to commercial units already in the market.

Transduction Mechanism	Sensor Type	Dimensions	Active Layer	Sensitivity Range	LOD	Operating Conditions	Response Time	Manufacturing Techniques	Reference
Traditional devices commercially available									
Optical	NDIR	L: 5–8.2 cm W: 3–5 cm H: 1.2–2 cm	–	0–5.000 ppm	2–20 ppm	4.5–20 V_DC_	20–120 s	–	[46]
Optical	PID (MiniPID 2)	Ø 20 mm C.V: 15 µL	–	0–40 ppm	1 ppb	10.6 eV lamp 3–3.6 V_DC_	8 s	–	[204]
Acoustic	SAW	L: 4.0 mm W: 1.0 mm H: 0.5 mm	6–30 nm (CNTs)	10–180 ppm	1–10 ppm	Room temp. f_r_ = 433.92 MHz noise: 3 kHz	2–4 min	–	[205]
Conductometric	Chemiresistor (MiCS-2714)	D: 5 mm × 7 mm H: 2.25 mm	MOS	0.1–10 ppm	50 ppb	High temp. (220 °C/50 mW)	12 s	–	[206]
Potentiometric	MOSFET (Z-900)	4.75 cm × 2.5 cm × 1.5 cm	MOS	0–50 ppm	0.1 ppm	High temp. 9 V battery power	<30 s	–	[207]
Microfabricated devices									
Optical	µPID	2.4 mm × 2.4 mm C.V: 1.3 µL	–	0–1 ppb	2–8 ppt	10.6 eV lamp 5–6 V_DC_	0.1 s	–	[48]
Gravimetric	SAW	20 mm × 20 mm	53.91 nm (CuO)	0–50 ppm	500 ppb	Room temp. f_r_ = 198.98 MHz noise: <300 Hz	10–90 s	Sol–gel	[66]
Gravimetric	CMUT	4 mm × 1.5 mm Ø_e_: 5.3 µm	50 nm (Polymer)	10–100 ppb	51 ppt	Room temp. f_r_ = 47.7 MHz noise: <2 Hz	<120 s	Direct wafer-bonding + local oxidation	[74]
Amperometric	RTILs	Ø_e_: 1 mm V_RTILs_: 2–8 µL	150 nm (Pt-TFEs)	0.1–2 ppm	20–110 ppb	Room temp. LSV (100 mV·s^−1^)	–	Electrodeposition	[90]
Conductometric	MOS-chemiresistor	13.4 mm × 7 mm (Ag-Pd IDEs)	Nanobricks (In_2_O_3_)	0.1–1 ppm	<100 ppb	Low temp. (50 °C)	114 s	Electrochemical anodization	[125]
Potentiometric	Polymer-FET	Au-elec. (30 nm; 20 µm × 1 mm)	20 nm (OSC-film)	1–25 ppm	1 ppb	Room temp. RH (45–70%)	5 s	Dip coating	[99]
Potentiometric	Bioelectronic-FET	–	12–15 nm (ORs + CNTs)	10 ppt–1 ppb	10 ppt	Room temp.	Real time (<5 s)	Photolithography	[180]

**Table 2:** Comparative analysis of macro- and microanalytical tools for the separation of VOCs.

Feature	Conventional GC Columns [212,214]	µGC Columns [229,240]	Microfluidic Channels [253,261,262]
Typology	Capillary	Capillary Chip based	Chip based
Geometry ^1^	10–100 m (L) 0.18–0.53 mm (∅)	1–3 m (L) 50–500 µm (W) 50–800 µm (H)	1–5 cm (L) 2–4 mm (W) 50–500 µm (H)
Selectivity ^2^	↑ L ; ↓∅	↑ L ; ↑ H/W	↑ L ; ↑↑ W/H
Layout	Circular spiral	Serpentine Circular spiral Square spiral Wavy Zigzag Radiator	Straight
Cross section	Circular	Square Trapezoidal Semicircular Circular	Square Circular
Coating materials	Polymeric films (PDMS) Inorganic sorbents (silica, Al_2_O_3_)	Polymeric films (PDMS) Carbon–polymer hybrids Inorganic sorbents (silica, Al_2_O_3_)	Polymeric films (Parylene) Pure metals (Au) Metal oxides (ZnO)

^1^ Length (L), width (W), height (H), and diameter (∅). ^2^ General premises to increase the selectivity or segregation power of each analytical solution.
